# Embedding Information into or onto Additively Manufactured Parts: A Review of QR Codes, Steganography and Watermarking Methods

**DOI:** 10.3390/ma15072596

**Published:** 2022-04-01

**Authors:** Muhammad Usama, Ulas Yaman

**Affiliations:** 1 Mechanical Engineering Program, Middle East Technical University, Northern Cyprus Campus, Guzelyurt via Mersin 10, 99738 Kalkanli, Turkey; usamamuhammad01@gmail.com; 2 Department of Mechanical Engineering, Middle East Technical University, 06800 Ankara, Turkey

**Keywords:** 3D printing, additive manufacturing, embedding information, QR codes, steganography, watermarking

## Abstract

The paper gives a detailed review of the approaches adopted for embedding information into/onto additively manufactured parts. The primary purpose of this paper is to review all the techniques adopted for embedding information, highlight notable trends and improvements in these works, and provide design and manufacturing pipelines to realize most of these works. It classifies these approaches into four different categories and summarizes the works carried out in each field. It also compares all the results in textual and tabular forms and then gives a detailed conclusion of the best works in terms of application and effectiveness. The four categories discussed are 3D QR codes, 3D watermarking, steganography and nonclassified methods. Lastly, it discusses the future extensions and potential improvements in the field of embedding information, while exploring manufacturing technologies.

## 1. Introduction

### 1.1. History and Basics of AM

Over the years, there have been many notable developments in technology-related fields, ranging from conventional machining to computerized numerically controlled machining (CNC) and direct numerically controlled machines. The field of additive manufacturing (AM) known as 3D printing, in its pioneering stages, is a result of experiments carried out on numerous technologies. The advent of AM began in 1984 when a researcher named Charles Hull, later owner of 3D systems, filed a patent for layer-by-layer manufacturing. His work was recognized as one of the leading works in the field of additive manufacturing, which was followed by another patent filed by a researcher named Scott Crump in 1989, later known as the founder of Stratasys. The duration of the patent lasted for almost 20 years, during which 3D printers were not accessible to the public. In 2009, after the patent expired, an era of democratization of AM began, starting with the concept of rapid prototyping. This concept involved manufacturing models to test their fit, form and function requirements in various engineering fields. Further developments took place and resulted in the manufacturing of end products that were ready for use. Therefore, the name 3D printing finally received the name of additive manufacturing.

Additive manufacturing, as the name implies, involves the addition of material layer-by-layer. It is exactly opposite to subtractive or machining methods and is based on volume modelling rather than the surface. A common generic AM process starts with the creation of a digital 3D model in a computer-aided design (CAD) environment. There are various CAD software tools facilitating the creation of 3D models. Once the model has been designed in the CAD environment, the 3D model file is converted into a format called stereolithography (STL), which approximates the surface of the model with triangle meshes containing the coordinates of the normal vectors and the vertices of the triangles. The STL format is the de facto standard, and there is a slow shift towards a file format with more advantages—the additive manufacturing file format (AMF). Once the file is converted into STL, it is loaded into the computer-aided manufacturing software (CAM) and slicing is carried out, producing machine codes. If these codes are generated within the CAD software directly, then it is known as direct slicing, and is preferable for complicated models. In the CAM software, numerous steps are taken such as setting the layer thickness, infill patterns, temperatures, power, supports and scaling, etc. Once these properties are outlined, a command to print is conveyed to the machine and the fabrication process starts. After the fabrication is completed, the part can be removed carefully, and the supports (if utilized) can be cleaned or dissolved, depending on the material used in the post-processing stage. The final part can then directly be put into use.

The advent of AM proved to be advantageous for many reasons. It enables the manufacturing of complex geometries with ease and reduces the lead time as well. It significantly reduces the assembly steps and the cost of transportation due to remote production. It can also be used for several materials and avoids wastage. Internal channels can be developed easily and can be used for generative design, leading to mass reduction. Despite the advantages, numerous issues need to be worked out, such as the presence of porosity, mechanical strength issues and manufacturing time. However, with the continuous improvements taking place in the field of AM, it is a crucial part of the fourth industrial revolution.

### 1.2. Digital Ways of Manufacturing and Methods of Embedding Information into/onto AM Parts

As mentioned in the paragraphs above, AM initially involves digital manufacturing using software such as CAD and CAM. AM is better put into practice when it is used for mass customization rather than mass production, because it enables the manufacturing of complex geometries and complicated products. Considering the initial stages of AM, there is significant potential for embedding information into/onto AM products, which can then be retrieved from the final fabricated models. The era of AM will have a remarkable impact on the future of manufacturing technologies, simultaneously opening different areas of research in this field. Information in the AM parts can be embedded in various forms and used for different purposes, ranging from object tracking to object authentication. Information embedded inside the AM part can also be used to make the AM part more interactive for the user. The focus of this paper is to explore major existing embedding technologies related to the AM parts and draw a comparison among them. The forms in which information is embedded onto/into AM parts can be divided into four major categories, namely QR codes, watermarking, steganography and other methods. The last category involves all those technologies presented and published that do not fall under the former categories.

QR codes have been in use for a long time since their development, and major research has been carried out on their 2D forms. Taking a step further, a 3D QR code is generated if it is embedded onto/into AM part using different types of processes. This 3D QR code is easily scannable using a mobile device and does not get affected by wear and tear, as it is part of the product itself. This QR can be used for object authentication, tracking and monitoring purposes. This paper makes a comparison between different types of technologies involving 3D QR codes employing various AM technologies. Similarly, the concept of watermarking 3D content, such as videos and images, has been tested and applied for a long time; however, the advent of AM, involving digital manufacturing, created multiple problems for safeguarding copyrights. Therefore, to solve the copyright infringement issues, multiple technologies of watermarking 3D models (digital and physical) were proposed. This paper discusses all the major technologies related to embedding watermarks of 3D models and compares each of them in detail. A simple watermarking technique involves creating a watermark and embedding it inside the digital model. The model is fabricated, and cameras are used to scan and retrieve the watermark, hence protecting the original creators. Steganography, a term rarely used, is a technique of hiding information into/onto AM parts and retrieving it using a secret key. In this way, information can be conveyed to the end users without being leaked to the public. Different technologies involving steganography in 3D models are presented and compared. Specifically for this category, possible AM methods are mentioned in detail as well. The last category involves the usage of technologies in embedding information onto/into AM parts not belonging to the other three categories. These include works published to make the AM parts more interactive, such as integrating optical fibers, pressure sensors and air pockets. The following section gives a detailed overview of the structure of the paper and its layout.

### 1.3. Outline of the Paper

Considering the advent of AM around the globe, many 3D models have been created by various designers and manufacturers, from designing at a digital stage to achieving a fully functional physical product. This led to several avenues opening into different areas of research linked to the field of AM. One such avenue deals with embedding information onto/into additively manufactured products. Various approaches have been adopted to embed information and our paper aims to discuss these approaches and the works carried out in a detailed manner. This paper classifies the approaches into four main categories and focuses on the description and comparison of the techniques proposed in all four categories of embedding information onto/into AM parts.

In the introductory part, the paper discusses the history of AM and different types of AM technologies linking their usage in the field of manufacturing. The second section is a thorough review of digital manufacturing linked with AM technology and the potential to embed information onto AM parts. This section discusses the four categories related to information embedding, along with their advantages. It starts with the 3D QR code approach, followed by watermarking 3D models, followed by steganography. Finally, it covers other information-embedding approaches. The subsections of this section discuss all the approaches employed in the field of embedding information into/onto AM parts. A basic pipeline, valid for each category, and the primary procedure are mentioned. Next, a table is given to compare all the AM-related properties of each work for each category. This table is further explained in detail and every paper is thoroughly discussed. The third section covers the future trends and applications perspective, highlighting the best work in each category while also pointing out the advantages of these technologies related to the fourth industrial revolution, as well as explicitly stating the scientific benefits. Finally, a concluding paragraph summarizes the paper and reiterates the main aim of this paper.

## 2. Approaches for Embedding Information into/onto AM Parts

This section includes a complete discussion of different works and technologies proposed in the field of embedding information into/onto AM parts. It draws comparisons between different works for each category and lists down their limitations and future extensions. Each section contains a table that compares different AM properties of the works proposed in each category. It starts with the QR technology being applied into/onto AM parts for various purposes, such as object authentication and tracking. In order to prevent copyright infringements issues, the next category describes the watermarking techniques for AM parts in detail. To hide the information and retrieve it, a technique called steganography is mentioned in the third category related to AM technologies. Similarly, the last category includes all the information-embedding methods in AM parts which do not fall in the upper three categories.

### 2.1. QR Code

**Figure 1:**
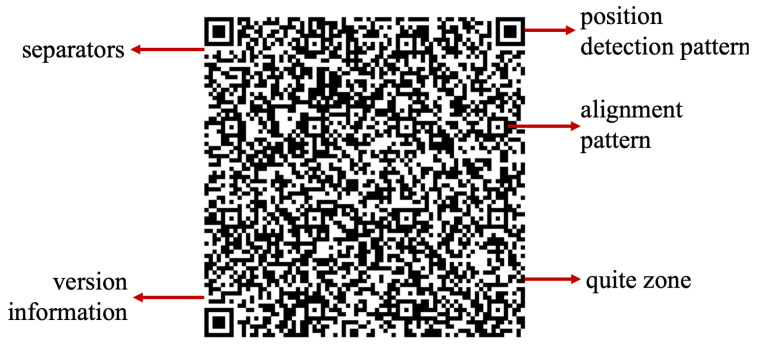
A QR code.

Each QR code, as shown in Figure 1, is composed of five main regions and features. Starting with the position detection pattern, this feature allows the reader to know about the QR code and its boundary. The alignment pattern ensures that the QR code can be read easily even in the condition of being skewed. The quiet zone basically refers to the empty region outside the QR code which helps the reader to differentiate between the area of the QR code from the outside elements. Version information gives the type of version of the QR code and separators improve the ability of the reader to recognize the finders more easily. There are other regions of the QR code as well, such as the error correction region, the remainder bit region, the data region, the format information and the timing patterns.

The QR code, which originated in 1994, was developed by Masahiro Hara, who was an engineer at Denso Wave. Before QR codes, a common coding system in use for tagging and identifying the products was the barcode system. With the shift from a single product manufacturing to a flexible manufacturing systems and production, the need for storing more information while occupying less space became crucial. Therefore, experiments were carried out to increase the data storage capacity of the barcodes for better efficiency in the industry. However, due to certain limitations of the barcodes, such as storing 20 alphabetic characters and coding in traverse direction only, research for creating a 2D code started. After numerous efforts, Hara, along with their team of engineers, was able to come up with a 2D code that had 3 position detection patterns, making it easier to be read by scanners [1]. This 2D code was able to store 7000 figures along with coding Kanji, Kana, and Hirigana characters. This 2D code was termed as QR (square) code and incorporated certain advantages compared with the barcoding system, such as being able to be read accurately and easily. Even if the QR code is stained, the error correction function allows the QR code to be read completely [1]. The technology of QR code keeps on evolving like other different technologies and has transformed from being printed onto a piece of paper to being embedded onto or into the additively manufactured parts. Due to the recent technological advances in the field of AM, product tracking has become one of the most crucial aspect; therefore, the usage of QR codes on AM parts has emerged.

**Figure 2:**
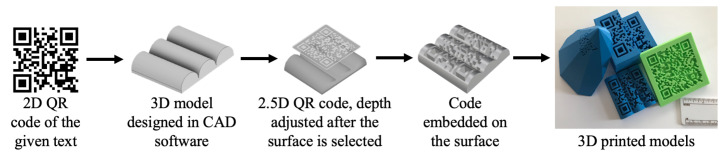
Design and manufacturing pipeline for embedding QR codes on the artifacts.

The basic procedure followed in most of the techniques proposed in the literature, given in Figure 2, starts with obtaining a 2D QR code which represents any type of information, such as a URL of a website or text. A 3D model is designed in a CAD environment and the surface on which this QR code is going to be embedded is selected. Once the surface-selection procedure is completed, different software can be used to adjust the embedding depth of the QR. The embedding procedure can be carried out using multiple techniques, namely grooving, carving or solid subtraction. The file can then be exported to CAM software, and different scaling, orientation and transformation procedures can be carried out, and requirements of generation of supports can be addressed. The model can then easily be fabricated using a 3D printer with the QR code embedded into the model.

#### Descriptions and Comparisons of the Methods

Table 1 compares different properties of studies on embedding QR codes onto the surfaces of additively manufactured products. It associates different works and research carried out in this field in relation to various properties. The columns in the table list all the properties, whereas the rows in the table compare the works carried out in this field.

As with the first implementation, the concept of 3D QR code was proposed by Kikuchi et al. [2], who suggested a different approach of the use of QR codes in a 3D environment. With the advancements in AM technologies, many concerns grew regarding the product authenticity, such as safety checks and tracking. They proposed a novel technique to embed QR codes onto freeform surfaces which were represented by B-splines. In their methods, black regions represented carved grooves and white regions represented the ungrooved surface. The method was carried out on four different models, manufactured using a fused-filament fabrication (FFF) printer. For all the models, the scanning time of the QR code was recorded to be more than 21.8 s. No post-processing steps were employed due to the accuracy and smoothness of the FFF printers. There were some limitations associated with this process. The work was carried out on a single B-spline surface in the presence of ambient lighting. The scanning angle was found to be less than 20${}^{\circ }$ and a single homogeneous material was used. This method fails if the materials used are either of highly dark or light colors. Moreover, the method has problems in the presence of directional light. This summarized method by Kikuchi et al. [2] marked the beginning of embedding QR codes onto the AM surfaces and gave the concept of 3D QR codes. Their method can further be extended for using materials that do not obey the Lambertian reflectance and further explore QR code techniques which are highly robust to changes in the illumination.

In the same year as the previously mentioned study, a similar work was carried out by Chen et al. [3]. Unlike the earlier one, this work mainly focused on embedding QR codes while maintaining the structural integrity (strength) of the part. Three different technologies are used in this work, namely FFF, material jetting (MJ) and direct metal laser sintering (DMLS). The model is a tensile test specimen on which the QR code is embedded. The QR code is first divided into five different parts and then it is embedded at different depths inside a cube placed inside the part. The code can only be scanned from the front side and testing is carried out for strength analysis using both the QR-embedded specimens and a simple specimen having no QR codes embedded inside. It was observed that there was no crucial difference between the strength of both specimens. After obtaining the image of the QR code using micro-computerized tomography, this image is further improved by going through a series of processes. The image is then read by mobile phones. There was no post-processing carried out compared with the previous study. Although this work embeds QR codes using different AM technologies, there are some drawbacks. Only straight surfaces were considered in this work. Moreover, porosity inside the parts can present a big issue while using the SLM technology. It also fails to read the QR code directly as image processing is a crucial intermediate stage. The process can be further extended to complex areas by carefully segmenting the QR code and adopting appropriate layer thicknesses.

A notable and novel QR-code-embedding approach was proposed by Wei et al. [4] using selective laser melting (SLM) technology. They identified several defects in the work proposed by Chen at al. [3], and the most important of them was the fatigue failure of the components due to the presence of voids and porosity. The use of a single material while building the QR code and dividing it into small voids can cause stress distribution and result in failure of the part. They proposed using multiple material AM which can overcome this issue by creating strong bonds between the main building material and the material used for filling the pixel voids after melting. Three different scanning techniques on three different samples were used. The first sample A had a QR code fully exposed on its top surface. The second sample B had a QR code whose two-third area was covered by 316 L layers, whereas the final sample C had the QR code completely covered by 316 L layers. It was found after a series of experiments that large atomic difference between the building and the tagging material results in better resolution and contrast. Another important finding was that the layer covering the QR code should be thin and ideally not more than 3 mm. This work proposes a novel technique to embed QR codes using a multiple-material SLM technique on flat surfaces. Unlike the work carried out by Kikuchi et al. [2], this work does not focus on the freeform surfaces and there is still some porosity—not suitable for aerospace applications. Moreover, complicated imaging techniques are required to obtain the QR code image, which makes it expensive and results in higher manufacturing costs. Despite the difficulties and shortcomings of this work, it proved to be a significant milestone for embedding QR codes in inaccessible areas of the metal parts. This technique can be integrated in the block-chain to assure certification of the part as well as recording its life cycle.

In addition to the fabrication of the metal parts with QR codes using various AM methods, consumer-level implementations were also accomplished. Yang et al. [5] proposed a novel method to produce 3D QR codes for similar identification and authentication purposes. They investigated that the QR code produced should satisfy three major things, namely connectivity, structural soundness and decoding robustness. Their experiments were divided into four parts. The first part was scanning using a mobile phone. The second part was scanning using different background colors. This was also a success—because the material was white polylactic acid (PLA), there was a significant contrast present. Thirdly, they used different 3D printing materials and noted down that the successful decoding was contributed to a significant background contrast. Lastly, they used different scanning angles and concluded that it was difficult to decode for angles greater than 30${}^{\circ }$. Unlike the work carried out by Kikuchi et al. [2], this technique can be applied in general manner to various models and is not limited to B-spline representations or CAD models. The QR code carving is carried out on the surfaces, unlike the works carried out by Chen et al. [3] and Wei et al. [4]. Moreover, there is no post-processing of the part. This work is limited by the use of contrasting background and scanning angles. It can further be extended for the functionalities of 3D QR codes, such as areas other than the product tracking and authentication, and on the disconnected components of the QR code.

There was another work in the field of embedding QR codes using self-shadows. Peng et al. [6] proposed to use self-shadows to carve out the black-and-white modules of the QR code on thin-shell surfaces and even on highly curved ones. These QR codes can easily be embedded using commercial-level 3D printing technologies. The main issues were the robustness, generality, printability and appearance of the QR codes. To minimize support structures and effects on the appearance and printability, the carving depth is optimized. The embedded QR code is scanned by using simple mobile phones. The criterion of successful decoding was set to be 3 s. The experiment was also repeated in several different lighting setups, and it was concluded that it became difficult to scan the QR code during noon time as the lighting gets strong. Additionally, it was found that the success rate decreased as the scanning angle increased above 50${}^{\circ }$. No post-processing was carried out during the experiment. The technique uses a single homogeneous material as opposed to the techniques proposed by Wei et al. [4] and Chen et al. [3]. It has the potential to be improved, because it can be further investigated in the presence of directional light. This work can also be extended to different AM technologies using various colored materials.

**Figure 3:**
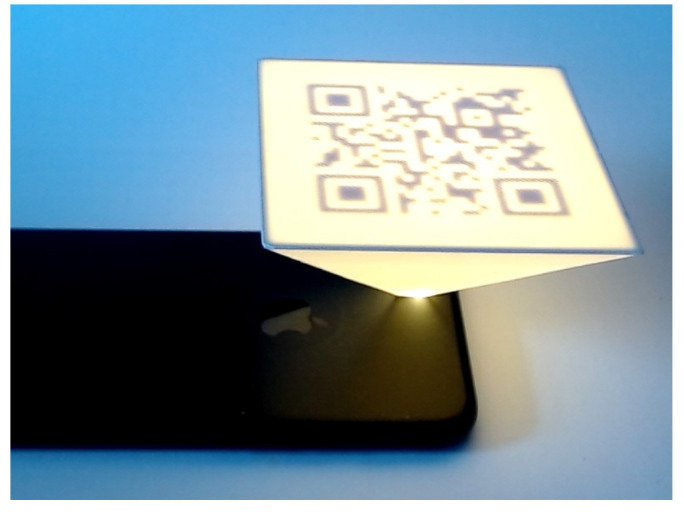
Hidden QR code at the base of the part.

There are other studies using the same technology (FFF) and the same material (PLA) to embed the QR codes onto the surface of AM parts. In one of these studies, Gultekin et al. [7] proposed an automated approach to embed QR codes onto the interior surfaces of 3D-printed parts. This work mostly focuses on hiding the QR code into the AM part which can only be read by a light source as shown in Figure 3. If the error correction factor increases above 30%, then the readability of the QR code is affected. The QR code embedded can easily be scanned using mobile phones. Moreover, there was no post-processing carried out after the part was manufactured due to the capability of the FFF 3D printer. This approach has its own limitations, for example, it only involves making use of a single homogeneous material and scanning can only be carried out with a background light source. Additionally, it requires a thin layer below the place where the QR code is to be embedded. It should be noted that the de facto file forming AM is STL and it is not suitable for storing information into the surfaces of the AM parts. An approach of direct slicing can be used to overcome this issue. Unlike the previous works [2,5,6], where QR was embedded on the outer surfaces of the AM parts, this work focuses on embedding the QR code on the interior surfaces. The absence of mention of scanning angles in this work makes the reader assume that the QR code was scanned from the front. Despite the limitations, the work carried out by Gultekin et al. [7] paves a significant pathway in exploring the functionalities of the QR codes in the AM field.

Following the previously mentioned works, Peng et al. [8] introduced a novel technique of embedding QR codes which resulted in with minimum alteration of the AM parts. The focus of this work was that the QR code was scanned using a directional light source. An important aspect of this work was to make sure that the QR code can be embedded onto the thin and hollowed structures without introducing holes or cracks on surfaces of the AM parts. Hence, the QR code embedded were of smaller size and shallow depth. In this work, the main issue of balancing unobtrusiveness, readability and printability is solved by using an optimization algorithm. The 3D printer employed in this process uses the technology of stereolithography (SLA) and the material is an opaque photopolymer. Compared with the works carried out in [2,5,6], this work mostly makes use of high-precision 3D printers, as fine features and minimum carving are required. Additionally, there are no support structures required in embedding the QR codes due to their small sizes. The success of decoding decreases as the ambient lighting conditions are increased and as the scanning angle was increased above 50${}^{\circ }$. However, it proved to be more robust than the previous works [2,5,6]. It was found out that the directional light angle range where the 3D QR code worked was within ±2.5${}^{\circ }$. There were certain limitations of this approach as well, because it was found out that the success rate dropped when the module size decreased to less than 0.9 mm. Moreover, the success of this approach decreased as the curvatures of the surfaces increased. The QR code can only be scanned in the presence of a directional light and post-processing was a requirement. Compared with [6], this work has superior optimization algorithm in terms of the visual unobtrusiveness and the carving depth. It can further be extended to optimize the position on the surface where the QR code is embedded and to increase the scanning angles.

Compared with the previous works of Kikuchi et al. [2] and Peng et al. [6,7], an improved method of embedding QR codes onto highly curved surfaces was proposed by Papp et al. [9]. In this work, they provide an improved method to prevent deformation of the highly curved free form surfaces during QR embedding. The carving method used in this procedure is the same one mentioned in the work carried out by Kikuchi et al. [2]. The focus of this work was to make sure that the QR code was successfully embedded onto the highly curved surface without further deformations. It was carried out by consumer-level 3D printers employing the FFF method with PLA material. Unlike the works carried out in [2,5,6,8], an optimum printing orientation is also determined in this work to build the inner parts precisely. However, this includes a drawback of including support structures, unlike in the work of Peng et al. [8]. The QR codes are scanned using conventional mobile phone scanners. It was found out that iOS was able to scan the QR codes better than the Android, but both took less than 5 s to decode. There was also no post-processing carried out on the manufactured parts. Four different models—namely a hill, stairs, a vase and a car—were used to compare this work with the works of Kikuchi et al. [2] and Peng et al. [6]. It was found that this work proved to be much better in embedding QR code onto the highly curved surfaces compared with the others. As the distance of the central projection decreased, the scanning of the QR code became difficult. There were certain limitations associated with this method as the QR code could only be scanned from a close or a far distance. Despite these limitations, Papp et al. [9] were successfully able to provide an improved method of embedding QR codes onto highly curved surfaces and their work can certainly be extended to determine the largest possible QR code size embedded around a specific point, as well as applying to other general meshes.

Reviewing all the works carried out in this category of embedding information onto AM parts, the work of Kikuchi et al. [2] laid the foundation of 3D QR codes using FFF, and the subsequent works were all based on the main principles laid out in this study. However, the limitation of the contrasting background, smaller scanning angle and the lack of experiments on various materials made this approach less usable. Contrary, the work mentioned of Chen et al. [3] explored multiple AM technologies, such as FFF, DMLS and MJ, and tried to embed QR codes at different depths, making sure the strength of the specimen was not affected. The major drawback resulted in the test being carried out on a straight part rather than a freeform surface. The intermediate step of image enhancement caused this technique to become extra expensive and the presence of porosity made it impossible to be used in the aerospace industry; hence, this technique required further room for improvement. To carry out improvements on this method, another technique was proposed by Wei et al. [4]. This focused on decreasing the porosity content and different AM technologies were experimented with by using multiple materials. However, 100% elimination of porosity could not be achieved, and the cost of scanning proved to be high as well. Nevertheless, this proved to be a significant milestone in embedding QR onto metal AM parts using SLM. Focusing on the low-level consumer 3D printers, the technique by Yang et al. [5] used FFF technology to embed QR codes onto any type of surface. However, only utilizing a homogeneous material, limitation of a prescribed degree of scanning angle and need of contrasting background proved to be a major shortcoming of this work. Despite its limitations, the work provided progress in this field of embedding QR codes. The study of Peng et al. [6] was aimed to increase the scanning angle using FFF technology and proved to be quite successful. However, it was limited by the time it consumed for carrying out multiple iterations and by physical environment. Nevertheless, a significant milestone in working out the issues persistent in the past techniques. Another FFF technique was proposed by Gultekin et al. [7] for embedding QR in the interior surfaces. Background light source was required for decoding and a homogeneous material was used in the study. Therefore, the technique did not focus on other AM technologies. Using the presence of directional light, and the technology of SLA, the study of Peng et al. [8] proved to be a significant milestone in decreasing the size of QR codes to be embedded. However, it became difficult to embed it onto highly curved surfaces. The last technology related to QR was proposed by Papp et al. [9] utilizing the FFF technology. The scanning time was reduced significantly and successful embedding of QR on highly curved surfaces was carried out. However, despite the advantages, the experiment was carried out only using a single homogeneous material.

Comparing all the works, the most applicable, time-saving, and cost-effective solution was the work proposed by Papp et al. [9], specifically developed for the FFF technology. With this technique, the successful embedding of QR on highly curved surfaces was possible with very low scanning times. Higher precision of the parts with embedded QR was also made possible using this technique. In the metal technology, the work proposed by Wei et al. [4] using SLM technology proved to be a significant milestone. It made use of multiple materials to increase bonding and decrease porosity while simultaneously focusing on the non-accessible regions of the metal parts making them easier to access and track.

### 2.2. Digital Watermarking and Copyright Protection Methods

**Figure 4:**
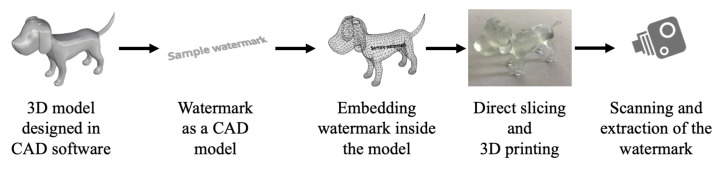
Design and manufacturing pipeline for watermarking inside the artifacts.

Digital watermarking is a technique used to prevent replication of the digital content and identify copyright infringement issues. It can be defined as a process which integrates digital data inside an object. With the growth of AM and an increase in the number 3D-printed parts, the need for digital watermarking has become more crucial in the recent years. Since AM begins with the design of 3D models in a CAD environment and ends with the fabrication on a 3D printing platform, the models produced can be reversed engineered easily and duplicates may cause a variety of copyright issues. Several watermarking techniques have been proposed over the years starting from embedding watermarks into the 3D meshes of a digital model to having watermarks inside the fabricated 3D objects. Watermarks can be divided into two types based on their perceptiveness—visible and invisible watermarks. The invisible watermarking technique can further be classified into spatial and transform-domain-based schemes, depending on the insertion space. Considering end applications, the watermarks are classified into two types, namely robust and fragile watermarking. Fragile watermarking involves the disappearance or changing of the watermark—once the object or the model is altered, it is invisible. The robust watermark remains inside the object or a model even though transformation attacks are carried out, such as scaling, rotating, filtering and resizing. The schematic in Figure 4 represents a basic design and fabrication pipeline of embedding watermarks inside AM parts. It starts with the creation of a 3D model in CAD software. Watermark information is then generated (with the help of a key sometimes) and is encoded inside the 3D mesh using different types of algorithms. The 3D model is then further exported to the CAM software where slicing of the model takes place. Sometimes direct slicing approach is used which embeds watermarking information into the fabrication instructions. A 3D printer fabricates the model with the watermark embedded inside. The detection process can be carried out using a camera or other types of similar equipment and watermark can then be decoded and extracted using statistical analysis and comparisons.

#### Descriptions and Comparisons of the Methods

Table 2 summarizes the major properties of various techniques proposed in the field of watermarking. A notable trend can easily be observed, the 3D models employed range from digital to physical ones. Coupled with other properties, the table briefly gives a glimpse of the advancements in the algorithms used for watermarking over the years along with the developing AM technologies.

Yeo et al. [10] suggested the first approach for generating watermarks inside 3D models in 1999. The watermarking scheme presented in this paper is the generalized form for robust and fragile watermarking. A 3D model is approximated via polygonal surfaces containing a vector of vertices and the surfaces are sufficient to describe a 3D model specifically for this technique. Yeo et al. [10] were able to achieve their goals of detecting minute changes to the original 3D model and counting out the number of alterations made inside the original model. However, their proposal was a preliminary one and required significant amounts of improvements and modifications in both the robust and fragile category. The proposed scheme was found out to be lacking in the situation where reordering of vertices was taking place. Their work comprised of detecting modification in a model without resorting to the original model and focused only on the geometry of the 3D object. This idea can further be extended to the information carried out by 3D models, such as textures, colors and normals, leading to a multiple-level verification.

The method of Yeo et al. [10] marked the beginning of research being carried out in the field of embedding watermarks in 3D models. Several works were 3D printed over the years by different researchers leading to a variety of techniques of embedding watermarks. One such method was proposed by Zafeiriou et al. [11], which was highly similar to the previous one in terms of the requirement of the original model at the end for detection purposes. A blind watermarking scheme is defined as the one which does not require the original model to be present at the time of detection. They presented a novel blind method for embedding a watermark that was robust to translation, scaling and mesh simplification. This method was the first blind method working effectively against mesh simplification and geometric attacks. The watermarking extraction procedure is carried out using the owner’s key and a threshold is determined earlier. The final decision of the originality of the model is taken by comparing the detection ratio with the threshold value. The difference in this work as compared with the previous one is the method of watermark insertion. This work makes use of the transform technique rather than the spatial one. Like the work of Yeo et al. [10], this method is applied and experimented on the digital models as well rather than the physical ones. It is a time-consuming method and requires high computational power. Other properties of 3D models can be studied, and the technique proposed here can be applied further to the areas of interest, such as color, texture and material.

To prevent counterfeiting of products manufactured using AM, and checking their authenticity, Aliaga et al. [12] proposed a method of designing and embedding a signature on the surface of a physical model to serve as a certificate of authenticity. They focused on embedding and testing using a genuinity-testing device. Contrary to the approaches mentioned above, this approach does not embed watermarks inside a 3D model; instead, it embeds a genuinity signature on the surface of the models. Compared with the approaches above, this approach also makes use of a camera to produce a digital image for verification purposes. However, the similarity of this approach [12] with the previous approaches [10,11] is the use of a key for verification. One of the main limitations of the method is that it requires space for the signature to be embedded. Moreover, the technique also requires approximate knowledge of both the manufacturing and the verification processes. It can further be applied on different types of objects, and manufacturing processes. Other feature spaces such as color and textures can be used as well.

Over the years, the techniques of watermarking have kept evolving at a fast pace. Another notable work was published by Suzuki et al. [13]. They proposed a copyright method for protecting the authenticity of the 3D models manufactured using AM technologies. Their work mostly focused on preventing illicit copies of digital data from being sold to the public without the original owner’s permission. They emphasized the significance of digital data in the age of 3D printers by stating that digital data is the first step in the creation of a physical model using a 3D printer. Hence, it is the one that is of considerable value and should be protected. A watermark in the form of fine structures is embedded inside a 3D model and read out using a technique called thermography. This method [13], as compared with the previous methods [10,11,12], does not deal with embedding watermark using a mesh of 3D models. Unlike conventional watermarking schemes, it mostly requires a physical model at the end to ensure its authenticity. Instead of employing algorithms, a simple method of thermography is carried out using a heat scanner. It makes use of a key in the form of a binary code that can be supplied to the consumer by the creator. This process has certain drawbacks as the technique of thermography is an expensive and time-consuming process. Moreover, it requires small cavities to be formed inside the fabricated object which can affect different mechanical properties such as strength and strain of the object. This method can be further extended to different materials other than PLA and testing can be carried out to ensure that the mechanical properties are not affected. Moreover, this method can be further refined to make the watermarks more invisible.

Macq et al. [14] provided an overview of digital watermarking schemes and analyzed how effective these methods are against safeguarding intellectual property rights. This paper focuses on a significant consideration—which was ignored in the previous papers—the effects of watermarks on the mechanical properties of the 3D models. It discusses some of the operations that a 3D model can face such as rotation, scaling, mesh simplification, translation, vertex or triangle index reordering, smoothing and cropping, as well as remeshing and topological modifications. The paper compares the non-blind techniques with the blind ones and states that such techniques ([12,13]) are useful in resisting these attacks one by one but further research is required if a scenario of combined attacks is presented. Similarly, blind techniques ([10,11]) are useful as they protect intricate features of 3D models however at the expense of security. Therefore, the blind techniques are not as robust as the non-blind ones. Future works can include more focus on a fully functional model of a complete part and scan process for different types of AM processes using various materials.

In another work, Hou et al. [15] proposed a robust watermarking technique based on a circular-shift-coding structure. This technique involves designing a model watermark that is robust to the distortions taking place during the 3D print and scan processes. As mentioned in the paper, there are high number of distortions taking place when a 3D model is 3D printed and scanned, hence the watermark embedded inside loses its originality and position. The authors summarized the number of attacks taking place during both the 3D printing and scanning processes. The attacks that take place during the fabrication are cropping, deformation of local coordinates, the stair-stepping effect (layer by layer process) and noise addition. The attacks that take place during the scanning process are remeshing, cropping, signal processing attack and noise addition. All these attacks are in the form of distortions. This watermarking scheme is advantageous over the non-blind methods ([12,13]) for several reasons. Firstly, this technique is applied on both the digital and the physical content and proved to be robust in all cases. In the physical case, its primary focus is to ensure that the distortions during the 3D print and scan process do not cause the watermark disappear. It also consumes less time and requires less computational power as opposed to the methods mentioned in [12,13]. Along with certain advantages, this method has also some limitations. The watermark must be 3D printed along the base axis of the model. If the watermark is embedded on a different location, it fails. In addition to this, information about the base axis is required during the scanning process for the watermark extraction operation.

Two years later, Hou et al. [16] decided to improve their earlier approach. They proposed a blind watermarking scheme which is robust against the attacks on both the digital and the analogue 3D models. The focus of the study is to make sure that the original model or information related to it is not required during the extraction process. As mentioned in their previous work [15], both 3D printing and scanning may cause significant number of distortions at various stages, hence affecting watermark embedding and extraction efficiency. Low-end 3D printers were used, and the technologies involved were laminated object manufacturing (LOM) and FFF. This method failed when it was applied on the high-resolution 3D printers, because it was difficult to detect the layers using a scanner. It is a bit different than the previous method [15], as it is a blind one, with no need of original model or its related information. There are certain limitations to this method as it requires the model z-axis and the 3D printing axis to be aligned. In the case of digital models, the watermarking scheme weakens against rotation attacks as it becomes difficult for the axis estimator to determine the original meshes. Moreover, this technique is limited to only cylindrical 3D models. It fails when presented with sharp edges. The method also cannot guarantee a perfect invisibility using current AM technologies. It can further be extended and improvements in processes such as remeshing can be carried out. The robustness and invisibility can be further improved by designing a 3D printing noise attenuator. CAD models—having curved surfaces other than the cylindrical ones—can be tested, and the algorithm may be modified and improved.

A notable method was proposed by Pham et al. [17] regarding the watermarks embedded onto the AM parts. It is composed of obtaining a 3D mesh and cut it into slices along the Z-axis. The conclusion of the originality is drawn by using statistical approaches between the original and the watermarked model. Three main parameters are evaluated—invisibility, robustness and performance. The work proposed by Pham et al. [17] has several advantages over the previous works. Compared with the works in [10,11], this method focuses on embedding watermarks and extracted them from the 3D-printed models. Hence, the technique proposed is an improved one and different from the conventional digital watermarking schemes. Similarly, compared with the works mentioned in [13,14], this method is less expensive and requires less time during the embedding and extraction procedures. Moreover, it does not require any key for the decoding process, unlike the previous works. It is found to be more robust than the works mentioned in [15,16]. This technique lacked in checking watermarking accuracy in physical models and more models should have been tested. Further research can be carried out using different materials and AM technologies.

Pham et al. [18] further improved their earlier research with the same intention of detecting copyright infringement issues. They proposed a novel technique of embedding watermarks using Menger facet curvature and K-mean clustering. The technique was tested on several different models and three parameters were checked namely invisibility, robustness and performance. The robustness of this technique was found out to be more than the methods mentioned in [15,16]. This is because, during the scanning process, distortions take place; however, the overall shape remains unchanged. Hence, no change in mean Menger curvature is observed. The performance of this method was also found out to be sufficiently better than the methods mentioned in [15,16]. This method had certain limitations of its own. It is based solely on the Menger curvature and, in the end, makes a comparison with the original model. This makes it a non-blind method which in certain circumstances can prove to be time consuming and resource exhaustive (if the original model cannot be obtained). The method can further be extended to other areas of other AM technologies. Different materials may be used to conduct the experiments using the same approach.

Similar research was carried out by Delmotte et al. [19] to embed watermarks to identify and authenticate the original 3D models from the counterfeits. They proposed a novel blind method of embedding watermarks using surface norm distribution. Compared with the previous works [15,16], this method is rotation-invariant, and hence does not depend upon the z-axis or the layered artifacts. The main benefit of this technique compared with [15,16] is that it is orientation-free and can be applied using high-quality 3D printers. Compared with the non-blind methods [12,13,15,17,18], this method is advantageous as it does not require the presence of the original model to make comparisons. The main drawback of the work is that it only focused on natural shapes. Designs prepared using CAD software are difficult to be watermarked as the flat surfaces decrease the imperceptibility of the watermark. This work can further be extended to different materials and various 3D printing technologies. Moreover, smoothing attacks can be investigated using the technique.

Peng et al. [20] proposed a technique involving noise signatures and designing a special authentication mark for extraction purposes. The researchers investigated two types of noises taking place during a typical 3D printing process, namely printing noise and observation noise. The experiment was repeated on several different 3D printers using various materials unlike in the works mentioned above [15,16,17,18,19]. Further analysis was carried out for checking robustness and the technique was found out to be robust against rotation and scaling attacks. It also had the capability of detecting polishing attacks as well as painting and waxing. This technique also proved to be advantageous as compared with other techniques proposed in [12,13]. The scheme has certain limitations as it requires certain flat patterns inside a 3D model which might not be possible in every case. The authentication marks embedded inside can distort the design. One more limitation is that the technique variably depends on the resolution of the 3D printers as well. Further research can be carried out to look for better authentication marks and mechanisms requiring as few modifications of the 3D model as possible.

Later, a notable technique was proposed by Delmotte et al. [21], in which they decided to prevent the circulation of counterfeit 3D products by using a similar watermarking technique. Their previous work focused more on embedding watermarks using surface norm distribution [20]. However, in this work [21], the novelty of their technique was that they embedded the watermark by modifying local layer thicknesses. The layer thickness is used because it is a constant feature and suffers minimum distortion. Therefore, it acts as a 1D carrier signal. This is used to embed the data. Different colors were used, and it was concluded that the robustness did not vary in response to a color change. This method had certain advantages over the previous methods. For CAD models having flat surfaces, this method can be used with low-cost and relatively simple equipment, unlike the methods in [12,13]. This technique has an edge over non-blind techniques [12,13,15,17,18] as it allows detection of watermarks without contact with the original model. Limitations to this technique are the preference for flat and smooth curved surfaces. Moreover, it was only applied on the FFF 3D printers. This work [21] can be further extended to exploring new extraction methods, improving the surface quality of the fabricated models, and reducing the visibility of the watermarks. Additionally, improvements in the error correction performance may be applied as well.

As mentioned in the introductory paragraphs, the 3D printing process is controlled by a G-code file which dictates each step of the print process. To prevent security issues of copyright infringement and tampering of the G-code file, a self-embedding watermarking process is proposed by Li et al. [22]. The procedure consists of three steps starting from embedding, followed by authentication and ending with the recovery of the G-code. This type of watermarking technique falls in the spatial category. The experiment was carried out on four G-code files using an FFF printer. This technique has certain advantages and can be considered as the first one in the field of embedding watermarks making use of G-code authentication. The difference between the work mentioned in [21,22] is that in [21], the local layer thickness is modified; however, no detail is paid to the originality of the G-code file. This work focuses on preventing direct attacks on the G-code file and recovering the original one. There are certain limitations to this technique as it cannot detect attacks that involve removing the command line from the G-code files. This technique can further be extended to different areas of AM technologies and various materials can be tested in the future.

The concept of watermarking 3D models began with the work of Yeo et al. [10]. They were able to successfully embed the watermark on a digital 3D model by focusing on the geometry (vertices) of the model. Although it is a pioneering concept in the field of watermarking, it required significant improvements. Continuing the work of Yeo [10], Zaferio et al. [11] proposed a blind watermarking technique for 3D digital models focused on the transform domain. However, it required greater computational power as it involved several iterations and proved to be only effective against geometric and mesh simplification attacks. Moving on from the digital to physical models, the technique proposed by Aliaga et al. [12] focused on embedding genuinity signatures on the surface of the AM parts. The AM technology used was FFF and the mechanical strength of the AM parts were not considered in the work. In addition, the requirement of the space on the model also proved to be a hindrance for this technique. With the continuous improvements taking place, the technique proposed by Suzuki et al. [13] proved to be significant as it involved FFF technology to embed fine domains inside the AM parts. The usage of thermography increased its cost of scanning, and the presence of cavities also affected the mechanical strength of the part. Nevertheless, it is an important step in the field of embedding watermarks into AM parts. Taking into consideration the distortions taking place during the fabrication and scanning procedures, the technique proposed by Hou et al. [15] was successfully able to defend itself against such attacks and proved to be applicable to other AM technologies, such as material jetting. One drawback was that this technique was base-axis-dependent and required the original model to be present. The same authors decided to make improvements in their approach using FFF and LOM technologies and presented a similar technique [16]. This technique was a blind one; however, it proved to be only useful against the cylindrical models. Another technique involving slicing parameters to embed watermarks in AM parts was proposed by Pham et al. [17]. The time consumed and the cost involved during the extraction was lowered significantly. However, only FFF technology was experimented upon which resulted in a lack of application for other materials. This research was further improved in terms of robustness with the work of Pham et al. [18]. With no distortions taking place in the printed part, a successful embedding of the watermark was carried out with little regard given to other AM technologies. Another work was proposed by Delmotte et al. [19] involving blind watermarking, having the advantage of orientation-free application. However, this work only proved to be applicable to natural shapes. The study of Peng et al. [20] proved to be very beneficial in this field as it involved testing its technique on several types of materials and various AM technologies. It proved to be effective against all types of attacks. However, it required a flat surface inside the 3D model and was highly dependent on the resolution of the 3D printers. The technique proposed in this category [21] involved using FFF technology and required flat and smooth curved surfaces for its application. The last technique, proposed in [22], is related to maintaining the integrity of the G-code file and preventing any tampering. It is presently the first watermarking technique for a G-code file and significant improvements are required in the proposed algorithm.

The work carried out by Peng et al. [20] proved to be significant as it involved testing watermarks on different types of AM technologies, such as SLS, FFF and SLA. It also made use of various materials and was able to effectively defend against the watermarking attacks carried out on the model. Moreover, it was also a blind approach, and hence did not require the original model to be present. The scanning procedure is a bit costly as it requires a microscope to detect the changes. However, it can be further improved and optimized with a combination of multiple technologies.

### 2.3. Steganography

**Figure 5:**
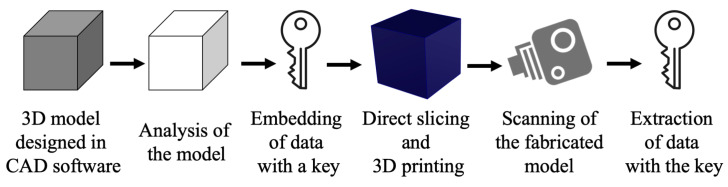
Design and manufacturing pipeline for steganography.

Steganography can be defined as the art of hiding communication inside a medium. Over the years, the media used to hide communication evolved from tattoos and invisible inks to digital content, such as an image, a file or a video. Democratization of 3D printing opened a new avenue for storing and hiding communication and a vast amount of research was carried out. The basic method in a steganographic system starts with the identification of redundant bits of a cover medium. The redundant bits can be defined as the bits which can be altered or modified without disrupting the integrity of the medium. A model called the stego model is created by replacing the redundant bits with the hidden message data. It is important to highlight the difference between watermarking and steganography here, as both techniques fall under the category of hiding information. A watermarking technique is aimed to make the watermark impossible to be removed without affecting the quality of the object, hence the technique mostly focusing on the watermark is highly robust. On the other hand, steganography focuses on hiding information that can get destroyed even slight modifications take place in a stego medium. Regarding steganography, two important definitions should be kept in mind, namely capacity and security. Capacity refers to the amount of information that can be hidden in a medium, whereas security refers to the inability of the eavesdropper to decode the hidden communication [23]. Figure 5 gives a general overview of the steganographic process for additively manufactured models.

The process starts with the creation of a model in a CAD environment. It is then approximated as a 3D triangle mesh and analyzed. Redundant bits are identified, and secret data is converted into a binary form using a key. Data are embedded inside the model in different ways depending on the technique employed. The model is later fabricated using a 3D printer and is then scanned using different 3D scanning techniques. The stego model is then analyzed for the hidden information and the communication is extracted using the same procedure of embedding but in reverse. The data obtained is in binary format which is then decrypted using a secret key.

#### Descriptions and Comparisons of the Methods

Table 3 lists major properties of various works carried out in the field of steganography on 3D models. Most of the works mentioned focus on digital models, whereas only one work concentrates on a 3D physical model. The table compares three important properties, namely capacity, robustness and security of all the works, and summarizes the limitations of each technique proposed. The following paragraphs provide a detailed description of each of the listed works.

Cayre et al. [23] proposed a scheme of steganography in digital 3D models using a substitutive procedure based in the spatial domain. This was the first time steganography has been used in 3D triangular meshes. The work was carried out to provide solutions for numerous applications such as side-information channeling, authentication and tamper proofing of 3D models. The scheme of steganography can be divided into two types, namely blind and non-blind. Blind scheme refers to the schemes which do not require the original model to retrieve the payload and vice versa. Similarly, in terms of signal processing, steganographic schemes can be classified into two categories, namely additive and substitutive. In additive technique, the payload is initially coded in a signal and is then added to the model, whereas the substitutive technique involves modifying the binary features for encoding payload. The procedure works by taking a cover model, payload and the secret key. Using these, the encoder produces a stego model. Similarly, the decoder takes the stego model and then using the secret keys extract the payload. Cayre et al. [23] mainly focused on the capacity and the security of the algorithm. It does not focus on robustness, hence it is vulnerable to local geometrical attacks, such as simplification and remeshing. This method can be further improved by employing multiple stencils to decrease the processing time. Moreover, the capacity of the technique can also be improved by using a generalized quantized index modulation (QIM) Method.

After the work of Cayre et al. [23], Tsai et al. [24] proposed a novel technique of steganography on 3D models using spatial subdivision technique. Their work falls under the category of substitutive blind technique like the one [23] mentioned above. The process begins with the construction of the binary space partitioning (BSP) tree. During the embedding procedure, three inputs, namely a cover model, payload and a secret key, are used. The embedding procedure ultimately produces a stego model. The extraction process works in a similar way starting with a stego model as an input. The robustness of the technique was checked, and it was found out that it had a higher effective rate against attacks, such as translation, rotation and scaling. This technique [24] proved to have higher robustness as compared with the technique [23] listed above. The capacity of the scheme also proved to be higher than the previously mentioned work [23]. One main limitation of this technique is that it employees a single stopping criterion, $V_{max}$, which limits the data hiding capacity. In the future, it would be better to employ numerous kinds of techniques for embedding and extracting procedures that can increase the data hiding capacity.

Later, Bogomjakov et al. [25] proposed a method of steganography that resulted in a distortion-free technique for polygonal meshes. This work was carried out to maximize the data hiding capacity while decreasing the complexity of the procedure. Unlike the works mentioned above [23,24], this work makes use of a method called permutation steganography, which provides an optimal embedding capacity. The decoding algorithm works similarly by comparing the mesh with the referenced mesh. The result of this work demonstrates a higher capacity than that mentioned in [23,24]. The capacity per vertex increases as the size of the model increases. The largest model involved in this experiment had the capacity of hiding 49.43 bits per vertex. It proved to be distortion-free and hence can be regarded as having higher robustness than the ones mentioned above [23,24]. Although this method is universal, as it employs a unique permutation steganographic technique and it can be used for any polygonal mesh, it requires an appropriate traversal technique to be chosen and rendering performance can be effected while permuting vertices and faces. The capacity of hiding data can be further improved by combining this technique with another scheme based on geometry. Although applicable to digital models, a further extension of employing the method on a physical model can also be studied.

**Figure 6:**
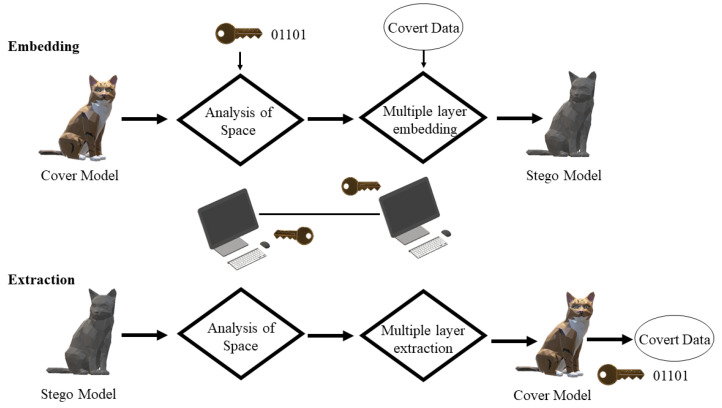
Embedding and extracting procedures [26].

In the following year, Chao et al. [26] proposed a novel steganographic model for 3D models with high capacities. The schematic is represented in Figure 6. Their approach is based on a multi-layer embedding scheme which involves hiding messages inside the vertices of a 3D mesh. As mentioned earlier, the focus of steganography is to ensure higher data hiding capacity and invisibility than robustness. This work takes capacity, reliability and security into critical consideration and considers robustness as a trivial parameter. The embedding procedure takes place in two steps. A similar procedure is followed but in reverse at the extraction stage as well. The vertex embedding order is determined by using the same method mentioned in [23]. According to the performance of the approach, it has a higher embedding capacity than the approaches given in [23,24]. It has a data hiding capacity of 21–39 bits per vertex which is smaller as compared with the technique given in [25]. In terms of robustness, it is a bit weaker compared with other approaches [23,24,25], since it cannot withstand attacks such as cropping, simplification and smoothing. In terms of imperceptibility, the approach makes sure that there is no visible distortion between a stego and a cover model. The proposed approach has certain limitations. These are the usage of perfectly smooth spheres and small-sized models which cannot be utilized as cover models. Usage of PCA hinders the robustness of the method and the approach cannot defend itself against transformation attacks. There is a need to improve vertex transverse list and to check efficiency on 3D physical models.

Later, Yuan-Yu Tsai [27] proposed an adaptive steganographic algorithm for 3D polygonal models with the help of vertex decimation. The main aim of the author was to focus on preserving important surface and shape features of the 3D models while embedding data inside them, hence producing less distortion and more imperceptibility. The algorithm makes a compromise between the accuracy of the complexity estimation and the embedding capacity. The experiment was carried out on eight different models and no error was found in the extracted message. The embedding capacity of this algorithm was found out to be 6.27–15.60 bits per vertex, which is less than [26] but greater than the others [23,24,25] mentioned above. In addition to the embedding capacity, this algorithm also focuses on the robustness of the models by carrying out a transformation process. Therefore, the robustness achieved by this technique is higher than the ones mentioned in [23,26]. Similar to the methods mentioned above, the algorithm adopts a blind approach for the extraction procedure. Important limitations of this method are that it requires a larger amount of time during the vertex decimation procedure and the embedding capacity estimation is solely based on the distance between the embedding vertex and the center of its referencing neighbor. This technique can be further improved by employing mesh curvature for better estimation. Optimizing the time required for the vertex decimation process can lead to quicker results. This technique can be further extended to point geometries and physical 3D models to test its efficiency.

Following the work of Tsai [27], Kaveh et al. [28] proposed a steganographic method of a high-capacity and low distortion 3D polygonal meshes using surfacelet transform approach. It was based on the transform domain and provides robustness to the 3D model. The experiment was carried out on a Stanford bunny model, and it was found that the maximum capacity achieved by the algorithm is 3 bits per vertex. Although this capacity is a bit low compared with other methods ([24,26,27]); however, it is the highest one in the transform domain. The capacity was higher than the proposed algorithm of Cayre et al. [23]. The robustness of the algorithm is also higher than [23,24,26,27]. Like all the techniques mentioned above, this algorithm works with the same blind extraction procedure. Its embedding capacity is very low compared with other methods in the spatial domain. The reason is that only the surfacelet coefficients that are related to the vertices are selected, hence making the 3D carrier sparse. Future research can be carried out using a Gaussian mixture model for better estimation and applying the proposed technique onto the fabricated models.

Later, Itier et al. [29] presented another approach for hiding data in 3D meshes using arithmetic coding. Their aim was to insert data one by one for each vertex as a function of the synchronization method of the Hamiltonian path. This results in increasing the data hiding capacity; however, it gives little importance to the robustness requirements of the 3D mesh. This experiment was carried out on a model of a bunny having 34,834 vertices and the results of the experiment demonstrated better imperceptibility than the ones mentioned above [23,24,25,26]. Moreover, it also proved to have a higher capacity of 3–24 bits per vertex than the methods mentioned above except for [26]. However, in terms of robustness, it provides very low robustness as compared with other methods [24,25,27,28]. Like all other methods, it follows the same blind procedure. The main limitation of the method is that it does not focus on the robustness requirements of the 3D model. Moreover, it requires slight movement of the vertices in the model resulting in low distortion; hence, it is not a distortion-free algorithm. The future extension of this method is to make the method more secure by employing a secret key in the path-building process. Moreover, robustness requirements of the 3D model should be further investigated, and experiments should be carried out on the actual 3D-fabricated parts.

After the work of Itier et al. [29], Li et al. [30] proposed a high-capacity steganography algorithm with adjustable distortion. The focus was to reduce the embedding distortion by confining the distortion within a very limited threshold. The algorithm simultaneously produces a high embedding capacity while adjusting the distortion of the cover model. The proposed algorithm was carried out on several digital 3D models and the results demonstrated a high embedding capacity while controlling the distortion rate. It was found to have the maximum embedding capacity of all the other methods mentioned above [25,26,27,28,29] having 90 bits per vertex of capacity. The robustness level was also found to be better than most of the techniques as [23,26,29]. It can withstand rotation, translation and uniform scaling attacks. However, it is vulnerable against attacks such as vertex reordering, local smoothing and local noise. Like all the previous approaches, this method also works on the same blind extraction procedure. Considering its limitation, the usage of PCA may fail to extract information if the right alignment of models with multi-axis symmetry is not found. Future extensions of this method may focus more on the robustness requirements and find out other strategies of embedding information. Moreover, AM parts can also be utilized to test the proposed scheme.

In another study, Suzuki et al. [31] proposed a technique of steganography inside AM parts by forming high-reflectance projections. Except for watermarking techniques mentioned above, this was the first time that steganography was carried out on actual 3D-fabricated parts. The procedure started with forming fine projections inside an AM part. These projections refer to the part which is filled and represent the binary information of 1. Similarly, the empty places inside the model refer to the parts which are unfilled and the binary information is 0. Hence the algorithm is dependent on the density difference of these areas. Images using a near infrared camera can then be obtained and information can be decrypted easily. The experiments were carried out using a 3D printer with two nozzles. One was for fabricating the model, while the other was used to fabricate the projections. The results demonstrated a higher embedding capacity which was estimated to be around 6.25 bits/cm${}^{2}$. The technique has lower robustness since any changes to the model will result in loss of projections and ultimately the hidden data. The main limitation of this technique is that by forming fine projections, it makes a compromise with the structural strength of the material, and hence can cause the failure of the part. The method can further be extended to other AM technologies and different materials can be employed such as ABS or using curved surfaces as models.

The main gap in employing steganography in the field of 3D models is the implementation of the algorithms on AM parts. Most of the research is carried out on 3D digital models and only few of them have been employed on 3D-fabricated physical models. It would be better to implement the techniques given above on real fabricated parts and check whether they can convey the information without disrupting the message hidden within them. Hence, major research is required in the field of steganography on fabricated AM parts.

The concept of steganography was proposed by Cayre et al. in [23], in which they were successfully able to hide information into a 3D digital model. However, the method failed in terms of robustness against geometrical attacks, as well as consuming considerable time. Working on the robustness requirements of steganography, the technique proposed by Tsai et al. [24] successfully resulted in increasing the robustness against outer attacks. However, due to a stopping criterion, the capacity of hiding information was limited. The technique proposed by Bogomjakov et al. [25] focused on increasing the capacity and robustness requirements. It had a higher capacity and robustness as compared with the previous works. On the other hand, the technique was limited due to the requirement of choosing an appropriate traversal technique. Later, Chao et al. [26] mostly focused on imperceptibility and no visible distortion was present between the stego and the cover model. However, it could not be applied to a perfectly smooth sphere and smaller sized models. Further improvements were carried out in this field and one such improvement as outlined by Yuan-Yu Tsai [27]. Robustness and capacity were further increased, but the process took a considerable amount of time. In the work by Kaveh et al. [28], robustness was the focus, and it was higher than the previous works, but it lacked in the embedding capacity as the usage of sparse 3D carrier provided hindrance in increasing it. Similarly, the work by Itier et al. [29] focused on increasing the embedding capacity but paid less attention on the robustness requirements. Additionally, slight movements to the vertices resulted in visible distortions on the models. The technique of Li et al. [30] had the highest embedding capacity and medium distortion as compared with the other works. Usage of PCA resulted in the failure in decoding if the right alignment of models is not obtained. The work of Suzuki et al. [31] was the first steganographic approach of AM parts depending on the density difference of the embedded projections. A higher capacity and robustness was found from the results of the experiment. Despite the advantages, the technique failed to focus on the mechanical strength requirements of the part.

In terms of the actual application of steganography on AM parts, the most suitable technique is the one proposed by Suzuki et al. [31]. The reason is that it not only focused on the robustness and distortion requirements but also focused on increasing the embedding capacity. Although it is an expensive approach, it has the potential to carry hidden data easily with minimum distortion. The approach of Li et al. [30] has the highest embedding capacity and robustness. However, it is carried out on a 3D digital model. Once the model is created, distortions may take place during fabrication and scanning processes which have not been identified in this study.

### 2.4. Other Methods

This section discusses different types of approaches developed over the years in the field of embedding information into/onto AM parts. All types of technologies mentioned here are completely different from the three categories discussed above and in no way share any similarity with them except for utilizing AM processes. The researchers embedded information inside the objects in various forms. These include embedding tags called infrastructs, which are based on materials and the information can be retrieved using Tetrahertz imaging. Moreover, this section also includes embedding of fiber optics to touch sensors making 3D parts more interactive, integrating AM parts with corrugated tubes and air pulses, forming fine cavities inside the AM parts representing information, embedding pictures inside the 3D parts, embedding tags for authentication and tracking purposes, and having different algorithms for detecting and extracting the embedded information. Table 4 represents the works carried out in this regard and compares their properties with each other.

#### Descriptions and Comparisons of the Methods

**Figure 7:**
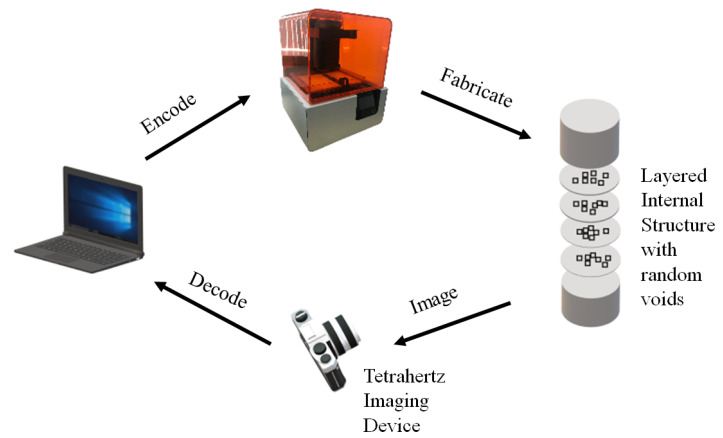
Infrastructs pipeline [32].

After the democratization of additive manufacturing, Willis et al. [32] proposed a technique of embedding material-based tags called infrastructs inside digitally fabricated parts using AM. A brief pipeline of the method followed in this work is shown in Figure 7. The procedure starts with encoding digital data on a computer. The user then specifies the region and to place the embedded tag. The tags were created for various purposes, such as location detection, object identification, object tracking and authentication. The depth and type of the embedded tag is decided and the model is fabricated on a 3D printer. They also proposed a technique of reading information using a Tetrahertz imaging system and the data is then decoded, respectively. These infrastructs were embedded inside the fabricated parts and were not visible to the naked eye. The algorithm works on the basic principle of the refractive index. The main limitation of this technology is that it is heavily dependent on the incident angle for the detection of signals, the depth of field is also narrow, and transmission is heavily dependent on the layer thickness. This technology can be further extended to materials other than ABS and various AM technologies. Mixing materials having different refractive indices may be proved to be useful in encoding and detecting large amounts of data.

Similarly, Hook et al. [33] proposed a technique for making AM parts interactive using wireless accelerometers. Small three-axis wireless modules are embedded inside the moving parts of the object and these modules are used as buttons and dials while employing a graphical interface. The experiment was carried out on a prototype of radio developed using FFF technology and PLA material. The main advantage of using such modules is that very little electronics knowledge is required for making the AM object interactive and only minor adjustments are required in the AM part. The main limitation of the method is that it is not suitable for devices that are in continuous motion such as game controllers and cellphones. Moreover, there is also no rotation around the z-axis of the accelerometer module. This technique can further be extended to materials other than PLA and various other AM technologies. Providing feedback to the users can also be explored while using these modules and two-axis controls can also be developed for game controllers.

To make the AM parts more interactive, Schmitz et al. [34] proposed a novel fabrication pipeline called Capricate for embedding touch sensors inside AM parts. Basic idea of the method is similar to the previously discussed study [33]. The process starts with a CAD model. Then, the user selects the type of sensor, the location and the size of it and as well as the surface of the object. The tool then uses the algorithm mentioned in [34] to automatically wire the sensors to the selected points and produce the file for 3D printing. The objects fabricated using Capricate in the experiment included an interactive wrist band, a touch-sensitive frame and a bracelet. The touch electrodes must be kept out of the sight and the overlay needs to have a height of one layer of 0.2 mm. The thickness of the layer greatly affects the sensing performance. The technique has several limitations as highly curved or smaller surfaces cannot be used for the embedding of these sensors. The low resolution of the printer limits the number and density of the electrodes embedded inside the object. Electrodes cannot be embedded on surfaces having a depth greater than 10 mm. This technique can further be extended to different AM technologies and materials. Touch on joy characters, braille patterns, numbers and icons can be distinguished using this technology.

Later, Stoll et al. [35] proposed a technique for integrating and embedding fiber optical sensors into AM parts manufactured using SLM technology. There are numerous advantages of having fiber optical sensors embedded in SLM parts. These sensors can be used to monitor structural health as well as to keep the inaccessible areas of the manufactured object in check. To test the functionality of the fiber, laser light was transmitted through the fiber and the results demonstrated successful integration and transmission. The fiber must be preprepared before it is integrated which has proved to be a laborious process. Moreover, there are still some issues of bonding on the lower side of the fiber with the coupon body which has proved to be troublesome. This technique can further be extended to other areas of AM as well as different materials can also be used for building purposes which can prove to be advantageous for the power generation industry.

Then, with the same goal of making AM parts more interactive, He et al. [36] proposed a technique of interactivity by having soft bellow like cavities inside the AM object. When the object is squeezed, air pulses travel through a corrugated pipe producing sound signatures. A microphone detects these signals and conveys them to a computer for interactivity. The main advantage of this technique is that it eliminates the need for additional sensors, electronic circuitry, and complex mechanisms for making AM parts interactive unlike the one mentioned in [35]. Moreover, the corrugated tubes embedded inside the cavities can easily be fabricated and are reusable. In addition, depth is also not a concern in this type of technique, unlike in [34]. Despite the advantages, the method suffers from limitations. These are the presence of ambient noise which affects the accuracy of the signal received. For slow pressing, sensing is not possible because of the weak air flow. Additionally, the technique fails to register continuous presses and can only detect discrete ones. It may further be extended to other materials and AM technologies while tinkering with different resolutions and fewer support requirements.

In another study, Suzuki et al. [37] proposed a novel technique for embedding information into AM parts. It started with the construction of fine cavities inside the fabricated object at the same time as the model’s body is being fabricated. The existence and nonexistence of the small cavities denoted 1 or 0 in ASCII format, respectively, and these were read out by using X-ray photographs. The cavities were embedded at different depths and the main aim of the study was to find out how much information volume can be stored inside the object using this technique. The experiment was conducted using a material jetting (MJ) type of 3D printer and the manufactured model was a black cuboid. It was found out that the extent of information that can be embedded inside one cavity ranged from 4 to 5 bits. Regarding the performance, regions deeper than 11 mm cannot be detected by X-ray photography. Moreover, due to the presence of these cavities inside the AM part, critical strength issues may arise. The technique may be applied on other AM technologies and materials.

**Figure 8:**
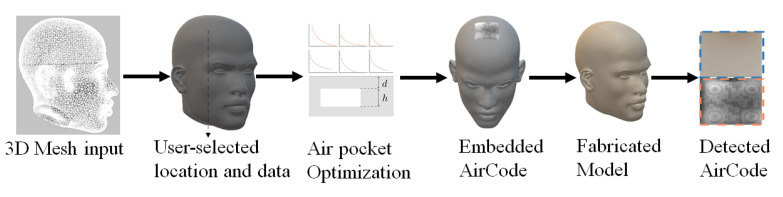
AirCode pipeline [38].

Li et al. [38] proposed an idea of embedding information in AM parts in the form of air tags. An air tag is composed of carefully designed pockets that are fabricated beneath the surface of the object during the fabrication process. Figure 8 represents the pipeline followed to encode air tag inside a fabricated object. The procedure starts with an input of a 3D mesh. The user specifies the region and the data to be embedded. The air pocket optimization is carried out and the program embeds the AirCode inside the digital model. The Information is encoded inside the air pockets in the form of markers and bits. The air pockets represent 1 while the filled solid part represent 0. The amount of information that can be stored inside the object using this technique can be at most 500 bits for a square tag of size 5 cm. Data are decoded using a light transmission source from a projector and the images are then captured using a monochrome camera. The results showed that decreasing the width can increase the amount of information stored but at the same time it makes the decoding process more difficult. Moreover, it assumes that material is homogeneous and semi-transparent, which might not be the same for all the materials. Lastly, air codes cannot be updated easily unlike the optical codes and hence, it cannot be replaced after the object is manufactured. Highly curved surfaces present challenges in embedding the air pockets as well. This method can further be extended to different areas of AM technologies and materials. Other computational methods may be used to determine the optimum depth of embedding and make the scanning process less time consuming.

In another approach, Nielsen et al. [39] decided to propose a technique called pic print which takes a 2D image and embeds it onto an AM part. The main aim of the study was to provide a new medium for the users to capture their memories. It makes use of AM lithophane approach which reveals pictures when it is lit from behind. The procedure includes three main steps namely conversion of the image to a height map, mesh generation and fabrication. The experiment was carried out by obtaining a grayscale image and converting it into a lithophane which is embedded on an AM pendant. This process is a bit time consuming. Moreover, it also lacks implementation on highly curved surfaces. The process can be further extended to different areas of AM and usage of various AM materials can be employed. Moreover, further automation may decrease the overall design and fabrication time.

Later, Tejada et al. [40] proposed a technique for blowing-activated tags. This work was carried out to make AM parts more interactive similar to the works mentioned in [34,36]. This technique works by blowing air into the cavities which in turn produces sound recognized by a computer. The experiment was executed on 48 different objects containing 830 blowholes fabricated using FFF 3D printers. It was found that as the size of the spheres and the tubes increases, the accuracy of recognition decreases. The procedure has some limitations as the type and amount of in-fill affects the sound produced from the blowholes. The procedure can further be extended to different areas of AM technology and materials. Further refinements on the cavities can provide better recording and interaction. This technology can be used for making interactive toys for children, as well as helping visibly impaired people.

Then, Maia et al. [41] proposed a technique for embedding optical barcodes on AM parts. They used a consumer-level FFF 3D printer which can produce two distinct types of materials, hence resulting in two different layers. The procedure starts with the creation of a digital model in a CAD environment, followed by the information to be embedded inside the tag and the 3D printing orientation. The code is decoded using a conventional mobile phone scanner using the algorithm mentioned in [40]. The experiments yielded satisfactory results and it was found that the mechanical strength of the fabricated parts with all three different methods was completely preserved. The procedure has its limitations, with complete occlusion and poor illumination resulting in decoding failure. The process of decoding is a bit slower than the conventional optical barcode scanning time. All the limitations provide an avenue for future research in this field. Moreover, further research can be carried out on embedding layer codes in metal AM parts as well as increasing the information embedding capacity.

Silapasuphakornwong et al. [42] proposed a technique for embedding information onto AM parts making use of the double-layered near infrared fluorescent dye. This work focuses on fabricating internal patterns containing fluorescent dye at two different depths which increases the amount of information being stored inside the AM part. The technique makes use of binary information and represents this by the existence or nonexistence of internal patterns. The patterns formed contain fluorescent dye in minute amounts. an FFF type of 3D printer is used to fabricate the model and infrared waves are then transferred to the model and the patterns emit fluorescence. This irradiated fluorescence is then captured using a camera which then displays an image of the embedded information. The formation of patterns inside the object can result in decreasing the mechanical strength of the part. This technique can further be extended to other areas of AM and materials. More research can be carried out in finding ways to increase the embedding capacity.

Following this study, Silapasuphakornwong et al. [43] took their research further and decided to fabricate objects with embedded barcodes on the curved surfaces. A similar procedure as the one mentioned above was used to fabricate small patterns representing barcode information onto curved surfaces. The experiments were carried out on a half sphere and a cylinder. Around the curved rims, the barcode images captured were blurry and not easily scannable. This technique can further be extended to other areas of AM technology and materials. The decoding and embedding process can also be made faster by applying new algorithms and increasing the sharpness of the images captured.

Another different approach was proposed by Dogan et al. [44] on a technology called G-ID in which information is embedded using various slicing parameters. The process of slicing is an integral and crucial part of the AM process, and this process does not change the geometry of the model and unique textures in the surface of the objects can easily be identified by a simple mobile phone. The labels corresponding to the unique slicing instances can be anything including a URL, a name, a text file, etc. Once the model is completed, the 3D model is then exported along with the G-code files. A mobile phone app is used and the camera is aligned onto the object’s outline. The instances are then decoded, and the label is retrieved. The larger the distance of the camera from the object, the more difficult it becomes for the camera to decode. The camera can decode the labels at certain angles and objects having smaller bases cannot be decoded using this technique. This work can be further extended to different areas of AM technology and materials which can result in better resolution. Moreover, further research can be carried out on increasing the embedding capacity.

Lastly, Uyan et al. [45] proposed a technique for AM tags employed for cast part traceability. This approach falls under the category of a semi-automated approach using both the AM process and sand molding inserts for identification and tracking purposes. The process involves the direct part making of sand castings employing AM mold inserts containing 2D codes scannable by machines. These tags are then converted into a 3D tag using a 3D printer using wax. Once the wax tags are completed, they are then combined with a wooden pattern which will be used to fabricate the sand mold. The wax burns away as the mold is poured inside the pattern. The experiment was carried out by 3D printing three different sized dots. After cooling, the codes were scanned using a mobile phone having a barcode scanning application. High contrast is needed for tags to be readable. Moreover, dot size also affects the readability of the code. This work can further be extended to other areas of AM technology and various materials can be used during the experiments. The codes may also contain information about materials, sand, operator, time of the process, etc., in the near future as well.

In regards to embedding information onto AM parts, different techniques were proposed which did not fall under the three categories mentioned in the previous sections. In one of these studies [32], a technology called infrastructs was proposed and ABS material was used to implement this approach. The transmission of rays during the scanning process was found to be heavily dependent on layer thickness, scanning depth and incident angle; therefore, it is a useful technology but it still needs further improvements. To make AM parts interactive, a technology using wireless accelerometers was proposed in [33], having a wide number of implementations employing FFF technology. The major flaw associated with it was that it is not suitable for AM parts subjected to continuous motion. A technology called Capricate was proposed by Schmitz et al. [34] for increasing interaction using FFF type of 3D printers. Although it is a great initiative, it could not be implemented on highly curved or small surfaces. Using SLM technology, Stoll et al. [35] focused on embedding optical fibers onto AM parts for checking parts’ health and tracking. The main limitation was the time-consuming procedure for preparing the fiber to be embedded. Moreover, some bonding problems makes it difficult to embed the optical fibers. Using the same goal of making AM parts more interactive, a technique was proposed by He et al. [36] using FFF technology. This technique was based on small cavities inside the AM parts. However, the limitations, such as surrounding noise, slowness and continuous pressing, hindered its usage in a wider range. Another work, published by Suzuki et al. [37], was based on small cavities placed inside the AM part. It was found out that the presence of cavities affected the mechanical strength of the part. In the work of Li et al. [38], a technology called the air tags was proposed using material jetting. It was later found out that highly curved surfaces presented challenges on the embedding algorithm and the decoding process was a time consuming one. Using the pic print technology by Nielsen et al. [39], a 2D image was embedded onto an AM part. However, it had the same problem as the previous technology. It was difficult to implement it on highly curved surfaces. Later, a technology called blowholes was proposed by Tejada et al. [40] which involved blowing air into the cavities present in the AM part making them more interactive. However, it was later found out during the experimentation stage that the type of in-fill patterns affect the sound produced from the manufactured part. Using the technology of optical barcodes, a work was proposed by Maia et al. [41] employing FFF technology. The limitations associated with it such as slow decoding, cross mixing contamination and poor lighting presented a hindrance in furthering its usage. Using the infrared technique, Silapasuphakornwong et al. [42] focused on embedding information onto AM parts employing FFF technology. However, similar to the works mentioned above, the presence of cavities can ultimately decrease the mechanical strength of the part. This research was further improved, and the same authors proposed to use this technology on curved surfaces [43]. Their technique suffered from a few limitations such as the sharpness of the barcodes embedded needs to be improved. A technology called G-ID was proposed by Dogan et al. [44] based on slicing parameters. Using an FFF printer, a label was embedded inside the AM parts; however, the camera angle determined the amount of information retrieved from the label. The work presented by Uyan et al. [45] was based on a semi-automated approach using AM and sand molding inserts. High contrast was required for decoding the information and hence presented some problems.

It would be difficult to carry out an analysis to determine which technology is better than the rest, as all of them are built on separate concepts and algorithms. All these technologies aimed to embed information onto AM parts in one way or another. They were quite successful despite the limitations and proved to pave the way for more research in this field.

## 3. Future Trends and Applications Perspectives

The primary aim of embedding 3D QR codes started with product authentication and tracking. Regarding this, numerous works were published by notable researchers, which proved to be highly useful in accelerating the growth of 3D QR codes. Despite the numerous limitations of each work, as stated in the manuscript, they had specific future extensions other than the initial objectives. There were notable future trends that could be observed in 3D QR codes, as significant research can be conducted in exploring various techniques to minimize the scanning time and increase detection accuracy. The need for a background light for detection may be further minimized by exploring different embedding techniques. Various layer thicknesses and curved surfaces can be experimented upon to detect the maximum embedding depth, which is detectable using mobile phone scanners. Significant research is also required in the field of metal AM technologies. With the extensions of QR codes being embedded into metal parts, the metal industry can flourish, and the life cycle of the parts can quickly be recorded. In addition to the metal industry, different polymers of various colors can be employed to improve the detection ability without a contrasting background. A notable significant future extension could be increasing the range of scanning angles and obtaining the smallest possible size of a QR code using optimization procedures that are not affecting the strength of the products. An example benefit of such extensions could be the use of 3D QR codes on AM medicinal drugs, enabling the user to read the entire prescription method just by scanning them.

In the field of watermarking, the primary aim of embedding these watermarks was to prevent the illegal distribution of the original products and to minimize the reverse engineering effects on the parts produced. There are specific extensions of the technologies produced in the field of watermarking aside from the detection of copyright infringement issues. The parts produced using PLA and ABS materials can be texted and experimented with different colors, and watermark information can be encrypted on the normals of triangular meshes. Moreover, using different AM technologies such as those dealing with metal manufacturing, such as BJ, SLM and DMLS, can be tested to make sure that the metal parts can easily be detected for their originality. Further refinement of watermarks can be carried out to make sure that the watermark is as invisible as possible and the mechanical properties of the manufactured parts are not affected due to their presence. One possible extension could be using a fully functioning scanning technology that detects and decreases the decryption time of watermarks. Various algorithms can be employed to make sure that the scanner detects the slightest variations or attacks on the originality of the part by observing changes in the watermarks. A significant extension includes the construction of the part while bringing as few changes as possible to the embedded watermark during the print and scan process. An all-in-one technology can be developed and distributed, which deals with embedding and detection of watermarks at low cost and consuming less time. This will prove to have extensive applications in the field of manufacturing and indirectly will relate to strengthening the global economies.

The field of steganography is not new, and has been around for centuries employing different media. The main objective of employing steganography in the field of AM is to enable side-information channeling and tamper proofing of 3D models. Although the objectives of steganography and watermarking are similar, there are noticeable differences among them. One significant difference is the persistence of watermarks in the case of tampering. The main issue faced in this field of steganography is the amount of research and experiments carried out on the actual 3D models. Most of the algorithms available and the ones mentioned in this paper focus on digital 3D meshes. It is a known fact that during the fabrication and scan processes, the stego (code) hidden inside the model can get distorted, hence losing its ability to be decoded. There is a need for an optimized algorithm that can decrease the number of distortions taking place and increase the coding accuracy. The time required to decode the stego model and retrieve the hidden information can also be reduced. Point geometries inside the model can be explored for information hiding purposes, and better approximation and error criteria can be developed. The usage of steganography can further be expanded and integrated with Industry 4.0. Several types of information can be hidden in plain sight and detected using various mechanisms, thus proving beneficial for manufacturing and military industries, civil services, and political communication.

The last category mainly describes the types of technologies employed in AM not belonging to the former three categories. Aside from using PLA and ABS, metal technologies can also be employed for embedding information into/onto AM parts. Different materials having different refractive indices can also be used and mixed with encoding and decoding data. In order to make the AM parts interactive, user feedback can be employed, and controls can be integrated inside the AM parts. One significant extension could be embedding braille patterns onto AM parts which will help the visually impaired people and can be extended to anything ranging from a key to a machine part. By embedding fiber electronics and tracking the mechanical health of the part, the structural integrity of the parts can be extended. Significant research is required in eliminating the requirements of supports and decreasing the resolution as low as possible. The algorithms can easily be automated to decrease the embedding and decoding times, making them time efficient and increasing the embedding capacity.

The article highlights the notable works carried out and explores the scientific benefits and usage for each of them. The works carried out in the above mentioned categories can be linked with numerous scientific benefits and can be extended to various industries. A notable use of the QR code can be connected with the pharmaceutical industry in embedding prescriptions into medicinal drugs and tablets. With the help of bio-ink being used in additive manufacturing technology, simple tablets can have the whole prescription procedure written in them with the help of easily scannable QR codes. This will significantly decrease the side effects of improper usage of medicinal drugs and help in minimizing danger to human lives. With the developments in AM technologies and numerous companies working towards bio-friendly inks, inconsistencies with dental fixtures have been eased by employing Zirconia based brackets. By manufacturing these additively, numerous design problems related to human teeth can be solved, and the instruction of proper installation can be embedded inside these brackets using 3D QR codes. The use of watermarks in AM can play a pivotal role in tracing and tracking counterfeit products. This usage can be extended further when manufacturing human organs using bio-ink becomes a reality. A watermark can be embedded inside the human organ to assure its authenticity and can easily be tracked to its parent company with the help of scanning procedures. This way, illegal human organ trafficking can easily be curbed and stopped. Notable companies are working on building spheroids and 3D cell structures using various bio-printer assortments, and the dream of achieving a 3D-printed, fully functioning human organ is not far away. Once the technology is successfully developed, the usage of embedding watermarks can prove very beneficial to the medical industry and help further and prolong human lives. Aside from the medical sector, watermarks can be used in the automotive and aviation industry to detect the originality of the additively manufactured parts being used in the body of automobiles and aircraft. By lowering the number of counterfeit and faulty products, the number of human lives lost in accidents can be decreased. The art of steganography cannot only be used to detect counterfeit products but also in covert communication as well. By embedding information into additively manufactured parts, intelligence can be shared from one place to another secretly through means of different products. This can prevent intelligence leakage and can easily help maintain security problems around the globe. Using the technologies associated with embedding information into AM parts, the structural health of the part can be significantly monitored, thus help us determine the functioning part’s life cycle and prevent further damage to the entire machine.

With the invention and development of several new polymers and other materials in additive manufacturing, the pace of work being carried out in the field of embedding information into/onto AM parts has accelerated. It is not surprising to associate these advancements as part of the fourth industrial revolution (Industry 4.0) as they are proving to be significantly valuable in industries and factories around the globe. The fourth industrial revolution deals with the innovations in controlling the industrial value chain and the idea of converging the real world with the virtual world to help achieve industrial smartness. The benefits of the industrial revolution not only include increased productivity but also encompass an increase in flexibility, quality and speed. With AM, the 3D printers and CAM software connected to the cloud open new areas in production, value creation and mass customization. Hence, with the inclusion of 3D printers, the factories related to manufacturing can be converted into smart factories with the ease of mass customization and bridging the physical and digital gap between the products. The concept of embedding information into/onto AM parts will significantly affect the entire value chain as it will enable tracking of the products from the beginning manufacturing to the final destination of the end customer. It will also decrease the authentication costs and simplify the tracking procedures. Moreover, it will also prove to have numerous benefits in protecting the copyrights and assuring genuineness. With the help of embedding information, be it QR codes, watermarking or steganography, personalized production will be heavily encouraged, simultaneously cutting down inefficiencies and intellectual theft and enhancing customer involvement with the manufactured parts. With the increase in customer involvement, customer loyalty will increase, which will result in the growth of innovation. By using cloud platforms, cutting-edge intelligence can be shared easily about the product as it can easily be tracked down to the manufacturer using the information embedded inside, while Industry 4.0 mainly focuses on mass production and customization with little human involvement, Industry 5.0 combines human and artificial intelligence in a workplace. However, this does not even begin to describe the benefits that it will bring to the manufacturing field. With the freedom of design responsibility given to humans, the boundaries of design will be pushed even further while 3D printers simultaneously work for manufacturing products. With the real-time data of the manufacturing product being transferred back to the designer, they can highlight better use of the product to the customer. This real-time data and usage information can be embedded inside the additively manufactured parts for the ease of the consumer, bringing considerable benefits to the future industries.

## 4. Conclusions

This paper reviews significant technologies and works proposed for embedding information into/onto AM parts. It classifies these works into four categories: QR codes, watermarking for protecting copyrights, steganography (the art of hiding information), and other methods of embedding information on additively manufactured artifacts. All the categories are thoroughly discussed and compared. The best technique for each type is then selected for implementation while keeping various parameters in mind. The paper’s main aim is to emphasize the developments taking place at a rapid pace in the AM industry and highlight significant findings that will result in better improvements. These findings will further progress the fourth industrial revolution, simultaneously accelerating the global industries towards the fifth industrial revolution. All the technologies discussed above will rapidly improve industrial development and ensure the betterment of human lives.

## Figures and Tables

**Table 1:** Studies vs. 3D QR code properties.

Studies	Technique	AM Method(s)	Minimum Feature Size	Material	3D Printer	Internal/External	Scan Software (OS)/Technology	Scan Hardware	Additional Processing
Kikuchi et al., 2018 [2]	Grooving	FFF	0.4 mm	ABS	Zortrax M200	External	iOS/Android	Mobile Phone Camera	No
Chen et al., 2018 [3]	Division and Segmentation	FFF, MJ, DMLS	FFF—0.178 mm; MJ—0.016 mm and 0.014 mm; DMLS—0.020 mm	ABS, Clear Resin, White and Black resin, AlSi,10Mg Powder	FDM-Stratasys Dimension Elite, MJ-Stratasys Objet30 Pro, MJ-Stratasys J750), DMLS (EOS M270)	Internal, between layers	Micro-Computed Tomography	Micro-Ct Scanner (SkyScan 1171, Bruker)	No
Wei et al., 2019 [4]	Embedding (Deposition and Melting)	SLM	316 L—50 mm; Cu10Sn—50 mm	Powder-316 L, Tagging Material-Cu10Sn	Multiple Material SLM developed at the University of Manchester	External and Internal	Infrared thermal imaging, X-ray imaging, X-ray fluorescence	Thermal imaging—FLIR Systems T650 sc.; X-ray imaging—Varian medical systems PaxScan 2530; X-RAY WorX GmbH, XWT-225-RAC. X-ray fluorescence analyzer—Thermo Scientific, Niton XL3t700 s.	Yes
Yang et al., 2019 [5]	Carving	FFF	0.4 mm	White PLA	HORI Z500	External	iOS/Android	Mobile Phone Camera	No
Peng et al., 2019 [6]	Carving (Shadows for scanning)	FFF	0.4 mm	PLA	HORI Z500	External	iOS/Android	Mobile Phone Camera	No
Gultekin et al., 2019 [7]	Solid Subtraction	FFF	0.4 mm	PLA	Ultimaker 3 Extended	Internal	iOS/Android	Mobile Phone Camera	No
Peng et al., 2020 [8]	Carving	SLA	0.1 mm	Opaque Photopolymer	UnionTech Lite 600HD	External	iOS/Android	Mobile Phone Camera	Yes
Papp et al., 2021 [9]	Embedding	FFF	0.4 mm	PLA	e Prusa i3 MK2.5	External	iOS/Android	Mobile Phone Camera	No

**Table 2:** Studies vs. watermarking properties.

Studies	Spatial/Transform	Fragile/Robust	Non-Blind/Blind	Use of a Key	Physical/Digital Model	Detection Level and Method	Material Used	AM Method(s)
Yeo et al., 1999 [10]	Spatial	Fragile	Blind	Yes	Digital	Beginner	-	-
Zaferio et al., 2004 [11]	Transform	Robust	Blind	Yes	Digital	Beginner	-	-
Aliaga et al., 2009 [12]	-	Robust	non-blind	Yes	Physical	High (usage of camera—Canon Rebel Xti 10 MP)	PLA	FFF
Suzuki et al., 2015 [13]	-	-	non-blind	Yes	Physical	High-Thermography	PLA resin	FFF
Macq et al., 2015 [14]	-	-	-	-	-	-	-	-
Hou et al., 2015 [15]	Spatial	Robust	Blind	Yes	Digital and Physical	High—3D scanning using Maestro3D MDS400	PLA, ABS, Resin	FFF and LOM
Hou et al., 2017 [16]	Spatial	Robust	non-blind	No	Digital and Physical	High	PLA	FFF
Pham et al., 2018 [17]	Spatial	Robust	non-blind	Yes	Digital and Physical	High—MakerBot 3D scanner	PLA	FFF
Pham et al., 2018 [18]	-	Robust	Blind	Yes	Digital and Physical	High—HP 3D-Structured Light Scanner Pro S3 SingleCamera	PLA	FFF
Delmotte et al., 2018 [19]	-	Robust	Blind	Yes	Physical	High—hand-held microscope imaging: Anyty 3R-MSUSB401	Evolvel 28, Future Resin 8000, DSM Somos 14120, Zr550	SLS, FFF, SLA
Peng et al., 2018 [20]	-	Robust	Blind	No	Physical	Medium—one-shot paper scanner Canon PIXUS MG3630	PLA using different colors	FFF
Delmotte et al., 2019 [21]	-	Robust	Blind	No	Physical	Medium—one-shot paper scanner Canon PIXUS MG3630	PLA using different colors	FFF
Li et al., 2021 [22]	Spatial	-	Non-blind	No	Physical	Medium—algorithm using average related difference (ARD)	PLA	FFF

**Table 3:** Studies vs. steganographic properties.

Studies	Model (Digital/Physical)	Spatial/Transform	Blind/Non-Blind	Capacity	Robustness	Security	AM Method	Material Used	Possible Method
Cayre et al., 2003 [23]	Digital	Spatial	Blind	Low—one bit per vertex	Low	High	-	-	FFF
Tsai et al., 2006 [24]	Digital	Spatial	Blind	Medium—three times the number of embedded vertices	medium-effective against similarity transformation attacks	High	-	-	FFF
Bogomjakov et al., 2008 [25]	Digital	-	Blind	Medium—1 bit per element	High	High	-	-	FFF, SLA
Chao et al., 2008 [26]	Digital	Spatial	Blind	High—21–39 bits per vertex	Very Low	High	-	-	FFF
Tsai et al., 2014 [27]	Digital	Spatial	Blind	Medium—6.27–15.60 bits per vertex	Medium	High	-	-	FFF
Kaveh et al., 2015 [28]	Digital	Transform	Blind	Low—3 bits per vetex	High	High	-	-	FFF, SLA
Itier et al., 2015 [29]	Digital	-	Blind	High—3–24 bits per vetex	Low	High	-	-	FFF
Li et al., 2017 [30]	Digital	Spatial	Blind	Very High—90 bits per vertex	Medium	High	-	-	FFF
Suzuki et al., 2017 [31]	Physical	-	-	6.25 bits/cm${}^{2}$	Medium	High	FFF	PLA	-

**Table 4:** Other AM-embedding approaches and properties.

Studies	Technology	AM Method	Material Used	Printer Used	Scanning Method	Scanning Equipment
Willis et al., 2013 [32]	Infrastruct (Tags)	FFF	ABS	Stratasys Dimension SST 1200es	Tetrahertz imaging	Picometrix T-Ray 4000
Hook et al., 2014 [33]	Wireless accelerometers	FFF	PLA	N/A	-	-
Schmitz et al., 2015 [34]	Capricate (Touch Sensors)	FFF	ABS	Ultimaker Original 3D printer	-	-
Stoll et al., 2016 [35]	Fiber Optic Sensors	SLM	316 Stainless Steel	Concept Laser M1	-	-
He et al., 2017 [36]	SqueezaPulse (Air Pulse)	FFF	PLA, ABS	MakerBot	Microphone	Audio-technica ATR3350
Suzuki et al., 2017 [37]	Cavities	MJ	black polymer	Objet 30	X-ray images	(inspeXio SMX-225CT
Li et al., 2017 [38]	Aircode (air pockets)	MJ	white polymer	Stratasys Eden260VS	Light images	Grey Grasshopper3 monochrome linear
Nielsen et al., 2017 [39]	Pic-print (Lithophane)	FFF	PLA	Ultimater 2+	-	-
Tejada et al., 2018 [40]	Blowhole (Activated tags)	FFF	PLA	Qidi Technology X-One, LulzBot Taz 4, and LulzBot Taz Mini	Microphone	microphone integrated in laptops and smartphones
Maia et al., 2019 [41]	Layercode (Optical barcodes)	FFF, SLA, MJ	PLA, Resin	MakerBot, MakerGear, and MJ, Autodesk Ember	Conventional Camera	Android/IOS
Silapasuphakornwong et al., 2019 [42]	Near infrared fluorescent dye (Internal patterns)	FFF	ABS	Mutoh Value3D MagiX 2200D	Near Infrared Camera	-
Silapasuphakornwong et al., 2019 [43]	Near infrared fluorescent dye (barcode patterns)	FFF	ABS	Mutoh Value3D MagiX 2200D	Near Infrared Camera	-
Dogan et al., 2020 [44]	G-ID (Slicing Parameters	FFF	PLA	Ultimaker 3, Prusa i3 MK3S, Creality CR-10S Pro	Mobile phone application	Android/IOS
Uyan et al., 2020 [45]	Digital Code (Semi-automated approach)	FFF	Wax	ProJet MJP	Mobile phone application	Android/IOS

## Data Availability

All the data is available within the manuscript.

## Abbreviations

The following abbreviations are used in this manuscript:

3D	three dimensional
ABS	acrylonitrile butadiene styrene
AM	additive manufacturing
BJ	binder jetting
CAD	computed-aided design
CAM	computer-aided manufacturing
DMLS	direct metal laser sintering
FDM	fused deposition modelling
FFF	fused-filament fabrication
LOM	laminated object manufacturing
MJ	material jetting
PLA	polylactic acid
QR	quick response
SLA	stereolithography
SLM	selective laser melting
SLS	selective laser sintering
